# Pressure Sensors for Measuring Tibiofemoral Contact Mechanics in Meniscal Root Repair: A Systematic Review

**DOI:** 10.3390/s25051507

**Published:** 2025-02-28

**Authors:** Khalis Boksh, Beibit Bashabayev, Duncan E. T. Shepherd, Daniel M. Espino, Arijit Ghosh, Randeep Aujla, Tarek Boutefnouchet

**Affiliations:** 1 Department of Biomedical Engineering, University of Birmingham, Birmingham B15 2TT, UK; d.e.shepherd@bham.ac.uk (D.E.T.S.); d.m.espino@bham.ac.uk (D.M.E.); 2 Leicester Academic Knee Unit, University Hospitals of Leicester NHS Trust, Leicester LE1 5WW, UK; arijit.ghosh@uhl-tr.nhs.uk (A.G.); randeep.aujla@hotmail.co.uk (R.A.); tarek.boutefnouchet@uhb.nhs.uk (T.B.); 3 Department of Trauma and Orthopaedics, Morriston Hospital, Swansea SA6 6NL, UK; beibit.bashabayev@gmail.com; 4 Department of Trauma & Orthopaedics, University Hospitals of Birmingham NHS Trust, Birmingham B15 2GW, UK

**Keywords:** tibiofemoral contact mechanics, contact area, contract pressure, pressure sensors, posterior meniscal root tear, biomechanics

## Abstract

Background: Tibiofemoral contact mechanics (TFCM) is an accepted biomechanical metrics for evaluating the meniscus in its intact, torn, and repaired states. Pressure sensors are increasingly used, with accuracy and repeatability influenced by test conditions, their design, and their properties. To identify factors optimising performance, we performed a systematic review of the literature on their use for measuring TFCM in posterior meniscal root tears. Methods: The Cochrane Controlled Register of Trials, PubMed, and Embase were used to perform a systematic review using the PRISMA criteria. As laboratory and surgical setup can influence sensor performance, we collected data on specimen preparation, repair techniques, hardware use, and biomechanical testing parameters. Results: 24 biomechanical studies were included. Specimen preparations were similar across studies with respect to femoral and tibial mounting. Single axial compressive forces were applied between 100 and 1800 N at varying flexion angles (0–90°). Tekscan (Boston, MA, USA) was the commonest sensor used to measure TFCM, followed by digital capacitive sensors and Fujifilm (Tokyo, Japan). Factors influencing their performance included fluid exposure, lack of adequate fixation, non-specific calibration protocols, load saturation exceeding calibration, damaged sensels and inappropriate pre-test conditioning. Conclusions: Understanding potential factors influencing pressure sensors may improve accuracy, area, and pressure distribution measurements.

## 1. Introduction

The posterior roots of the menisci play a critical role in preserving meniscal function and the distribution of axial load across the tibial plateau [1,2,3]. Posterior meniscal root tears (PMRTs) compromise the circumferential integrity of the meniscus and prohibit the generation of hoop stress. In doing so, the chondral surface is susceptible to injury upon loading, and can subsequently degenerate [4,5,6,7]. Therefore, it is strongly recommended to repair these tears in appropriately indicated patients [8,9]. Tibiofemoral contact mechanics (TFCM)—in particular, contact area and pressure—provides a form of measurement for chondral injury, and is an accepted biomechanical metric for the evaluation of the menisci in its intact, torn, and repaired state [10,11]. Knowledge of the patterns of TFCM in these conditions will determine the approach one takes to restore knee joint function [12]. Pressure sensors are increasingly being used to quantify contact area and pressure [13,14,15,16,17,18,19,20,21,22], with importance placed on appropriate and correct utilisation, as such outcomes in biomechanical research can affect clinical practice and patient care. For instance, the performance of the sensors depends on the test setup and the laboratory conditions in which they operate. This includes their exposure to fluid, temperature settings, and humidity; the ease of insertion within the joint; their calibration sequence; and how the tibiofemoral joint is mounted to ensure the sensors are securely fitted for analysis [11,13,15,17,19,20,21,22]. The sensor accuracy, to include repeatability and bias, is influenced by its inherent design and properties. Therefore, to properly interpret studies using these techniques, it is crucial to understand the behaviour and limitations of the sensors within this context. In this respect, we performed a systematic review of the literature on the use and applicability of pressure sensors for the measurement of tibiofemoral contact mechanics following repair of posterior meniscal root tears.

## 2. Methods

### 2.1. Literature Search

A systematic review was performed and reported according to the standards of the PRISMA (Preferred Reporting Items for Systematic Review and Meta-Analyses) criteria [23]. Searches of Cochrane Controlled Register of Trials, PubMed (MEDLINE), and Embase were conducted from the inception of the databases to 15 January 2024, and repeated on 10 November 2024 for an update of the literature. Both dates are illustrated in Tables S1 and S2. The Boolean search items included (‘posterior horn’ OR ‘root’ OR ‘radial’) AND (‘meniscus’ or ‘meniscal) AND (‘tibiofemoral contact’ OR ‘contact pressure’ OR ‘contact area’). No restrictions were made on language, and efforts were made to obtain translated versions of all included studies. Restrictions were not placed on the date of publication or the journal. All relevant articles and reviews were examined for further relevant citations.

### 2.2. Eligibility Criteria and Outcome Measures

All biomechanical studies on cadaveric or animal knee joints that used pressure sensors to measure tibiofemoral contact mechanics (to include contact area and pressure) following repair of posterior meniscal roots were included. No limitations were placed on the type of repair (i.e., transtibial pull-through (TPR), suture anchor repair (SAR), side-to-side repair, all-inside repair), and studies with treatment for concomitant injuries were also included. This includes, but is not limited to, anterior cruciate ligament reconstruction (ACLR), meniscotibial ligament tenodesis, and open-wedge high tibial osteotomy (OWHTO). Whilst these additional techniques could potentially confound the analyses from the sensors, this review ensured interpretation of the sensors was confined to the sub-groups where only a root repair was performed. Exclusion criteria included computational-based studies, editorial letters, case reports, technical notes, and expert consensuses and abstract-only studies.

### 2.3. Study Selection and the Assessment of Quality of Studies

Two authors (Authors 1 and 2) independently reviewed the titles and abstracts, after which relevant papers were reviewed in full by each author independently. Those that met the eligibility criteria were chosen, with discrepancies highlighted and reviewed by senior authors (Authors 5, 6, and 7). The same 2 authors independently assessed the methodological quality of the biomechanical studies using the Methodological Index for Non-randomised studies (MINORS) tool (Table S3) [24]. The risk of bias of the included biomechanical studies was assessed and reported by the same 2 authors in accordance with the Risk of Bias in Nonrandomized Studies of Interventions tool (ROBINS) [25] Each item was judged according to high, moderate, low, or unclear risk of bias. Studies were deemed to have the highest risk of bias if they scored a high or unclear risk of bias.

Furthermore, the final level of certainty of evidence across all studies was assessed using the Grading of Recommendations, Assessment, Development, and Evaluation (GRADE) tool. Five domains were assessed to include risk of bias, inconsistency, imprecision, indirection, and publication bias [26]. Rating of evidence was high, moderate, low, or very low. High certainty of evidence implies the evidence is very likely to represent the truth, and low certainty and below indicates that the evidence is unlikely to represent the truth.

As laboratory and surgical setup can potentially influence the performance of pressure sensors, we collected data on specimen preparation, repair techniques, hardware use, biomechanical testing parameters, and the conditions. Where possible, further data were collected on the design and properties of the sensor.

### 2.4. Data Synthesis

Continuous-variable data were reported as mean ± standard deviation from the mean unless otherwise specified. Categorical-variable data were reported as frequency and percentage. Of note, when data were presented, it was ensured the statistical tests for the included studies were adequately justified; that is, parametric tests were performed for studies comparing mean differences between two groups (e.g., two-tailed independent *t*-test), where normal distribution of data with equal variance was assumed, and non-parametric tests (e.g., Mann–Whitney U test) were performed for studies in which non-normal data distribution with unequal variance was assumed. In addition, studies comparing mean differences between three or more groups were analysed to ensure parametric tests (e.g., ANOVA) were only performed when the assumptions of normal data distribution with homogeneity of variances were met, with pairwise post hoc comparison tests (e.g., Tukey) to identify groups that differed from one another. Furthermore, it was ensured that non-parametric tests (e.g., Kruskal–Wallis) were only performed when no assumptions of the data distribution were made, with adequate post hoc comparison tests (e.g., Dunns method). A value of *p* < 0.05 was considered statistically significant unless otherwise noted.

## 3. Results

**Figure 1:**
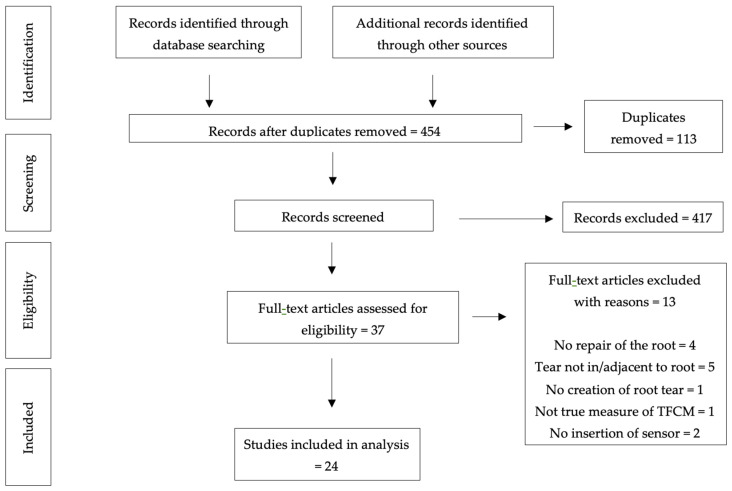
PRISMA flow diagram for study selection.

Thirty-seven full-text articles were reviewed. Thirteen studies were excluded based on the eligibility criteria (Figure 1). Twenty-four papers were therefore included in this systematic review [1,3,21,27,28,29,30,31,32,33,34,35,36,37,38,39,40,41,42,43,44,45,46,47]. The mean rating of all studies using the MINORS tool was 20.5 ± 1.35 points (of a maximum of 24 points, 85.4%; range, 19–22 points) (Table S4). A common reason for point deductions was item 5 (unbiased assessment of the study endpoint), as the observers were unblinded to the study endpoints and therefore may have influenced the results. This can potentially distort the true effect being measured, reducing the validity of the impact of the exposure being studied. The same reason was also noted following ROBINS assessment (Figures S1 and S2), whilst the certainty of evidence using the GRADE criteria was high across all studies.

### 3.1. Biomechanical Characteristics

In total, there were 235 testing specimens, of which 187 were in cadaveric and 48 in porcine knees. Seventeen studies measured tibiofemoral contact mechanics following the repair of a medial meniscal posterior root tear (MMPRT) [1,3,21,27,28,29,30,31,35,36,37,39,40,41,42,44,45], of which two performed additional treatment for concomitant injuries to include meniscotibial ligament (MTL) tenodesis and OWHTO [31,41]. The remaining seven studies were for lateral meniscal posterior root tears (LMPRTs) [32,33,34,38,43,46,47], and two studies performed either an ACL reconstruction or created in addition a meniscofemoral ligament (MFL) injury [32,33]. The baseline characteristics are described in Table 1.

Bone and specimen preparation were similar across most studies, including storage, dissection, preservation of knee-stabilising structures, and mounting on the testing machine. Despite the application of a single axial load across most studies, its range varied between 100 N and 1800 N in cadaveric knees, with 1000 N being commonly used. For porcine knees, this ranged between 200 N and 1500 N. Transosseous pull-through repair (TPR) was the most common repair performed for posterior meniscus root tears. Flexion angles that were commonly used were 0°, 30°, 60°, and 90°. Although this can affect the pressure on the joint surfaces, the consensus in most studies (twenty-one) was that the surgical repair of root tears restored tibiofemoral contact mechanics to that of the intact meniscus at most of these angles, and these results were similar when compared across studies [1,3,21,28,30,31,32,33,34,35,36,37,38,39,40,41,42,43,44,45,46].

### 3.2. Pressure Sensor Application and Insertion

Eighteen studies used the Tekscan pressure sensor (Boston, MA, USA) to measure tibiofemoral contact mechanics. The remaining studies used either Fuji Prescale films (Fujifilm, Tokyo Japan) [1,28,39] or digital capacitive sensors (Munich, Germany) [29,32,35]. The application and insertion of the pressure sensors are described in Table 2.

Capsular arthrotomies in the anterior and posterior meniscotibial ligaments were generally undertaken to position the sensors beneath the meniscus. Only three studies placed the sensors above the meniscus [27,35,44]. All but one study preserved the cruciate and collateral ligaments. This individual study sectioned the medial collateral ligament (MCL) for Tekscan insertion [27]. Fourteen studies secured the sensors within the knee, either with suture anchors [30,33,37,38,40,46], with screws [31,41,43,45], or securing them to the meniscocapsular junction [22,28,44,47]. Sensor position was confirmed during biomechanical testing either by disarticulating the femoral osteotomy initially created [30,31,33,34,36,38,40,47] or by cross-referencing its position with a standardised reference point [1,32,46].

Fujifilm was packed in a fluid-proof packet, preventing exposure to saline before and during testing. Tekscan was soaked in saline prior to testing in only six studies [33,37,38,40,41,45]. However, most soaked the sensor during testing to prevent shear forces and soft-tissue desiccation [21,27,30,33,34,36,37,38,40,41,44,45,47]. Five studies provided precautions to avoid measurement error in the presence of saline [30,33,37,38,40].

To deal with potential sensor damage or wrinkling, a new Tekscan sensor was generally used in the relevant studies [21,27,30,33,34,37,38,40,43,45,46]. Only four of these studies accounted for potential damage from repetitive loading on the sensors. This was by replacing false measures by means of the surrounding sensels [33,37,38,40]. However, none of these studies analysed the specific mechanism of damage.

A new Fujifilm was used for every test, whilst the same capacitive sensor was used throughout testing.

Five studies with Tekscan performed pre-tensioning [21,27,44,45,47], and two with capitative sensors [29,35]. For accurate Tekscan readings, two studies reported on their maximum detection limit [3,36], and three provided meticulous calibration to establish a baseline reference of 0 [21,44,45]. This ensured all sensors were activated and ready for data collection. Tekscan was frequently re-calibrated, either between tests [21,35,42,43,44,47] or with a new knee specimen [27,30,33,37,38,40,41,45,46] No re-calibration was required with the capacitive or the Fujifilm sensors.

## 4. Discussion

The purpose of this systematic review was to help researchers in making informed decisions regarding the applicability and limitations of pressure sensors in measuring tibiofemoral contact mechanics following repair of the meniscal root. Our review demonstrated that, qualitatively, surgical repair recreates the function of an intact state, and that a variety of pressure sensors can detect such change.

The piezoresistive Tekscan Pressure Sensor (Tekscan, Boston, MA, USA) was commonly used in the included studies. It is widely used in pressure and force detection and has been validated in several studies [48,49,50]. It allows for continuous data collection throughout several load configurations and dynamic simulation of joint movements in vitro. However, potential limitations to data accuracy involve several factors. These include crinkling or damage to individual sensels following repetitive manipulation and shear loading, load saturation exceeding calibration, output changes related to its placement and liquid exposure, and whether it is appropriately conditioned prior to testing [21,40]. During liquid exposure, the load output diminishes with time, with 90% of this decline mitigated by sensor exposure to saline for 48 h [51]. Therefore, saturating the sensor prior to loading improves the accuracy of the output recorded over time, reducing the likelihood of requiring post hoc data collection [51]. Six studies provided evidence of pre-saturation [33,37,38,40,41,45]. However, despite this, linear decline in mean total load outputs over several data captures for each knee is possible. Therefore, the acquired data should be normalised with the use of a measured linear rate of decline. This was undertaken in five studies [30,33,37,38,40]. Alternatively, one can prevent the output drift by isolating the sensor with a plastic film or Teflon tape [18,19,22,51]. Teflon, however, can potentially triple the thickness of the original 0.1 mm-thick sensors, inducing a 10–26% increase of contact pressure. Therefore, modifications that increase the thickness should be avoided [52,53]. However, polyurethane plastic film is 0.01 mm thick [18,52], which may potentially have a negligible effect on TFCM following repair of the meniscal root. This requires further investigation. However, a potential solution in dealing with the spurious results from sensor thickness, regardless of the technological design (resistive or capacitive), is to pre-tension the sensor first. This will remove the compressive pressure caused by its thickness prior to application of real compressive force. This was performed in seven of the twenty-one suitable studies [21,27,29,35,44,45,47].

Measurement accuracy relies on the secure attachment of the sensors. Anderson et al. reported that not all measured parameters could be evaluated with shifts in sensor position [54]. Fourteen studies in this review prevented this from occurring through securing the sensor on the tibia. Overestimation of the contact area and stress can confound the results when a shearing force is experienced within the joint. This was limited through keeping the sensors moist within the experiments and preventing the knee from over-constraining within the biomechanical setup.

Though the Tekscan sensor is reusable, its lifetime is greatly influenced by repetitive manipulation, testing, and subsequent wear following load. Damage to individual sensels can lead to erroneous contract stress and area measurements, identified from false pressure readings during no load. This can be minimised through periodic calibration checks, changing sensors, or replacing the erroneous sensel value with the mean of values reported by the surrounding sensels, as has been done in previous studies [17]. This was reported and performed in four of the included studies [33,37,38,40].

A potential factor that may affect sensor accuracy is the extent of knee disarticulation and soft-tissue dissection. Martens et al. reported that femoral condyle osteotomy for sensor visualisation and coronary ligament incision for sensor placement had no effect on the TFCM of the knee [55]. The minimal differences between the osteotomised joint and the osteotomised joint with an incised coronary ligament indicated that the ligament does not involve the transmission of compressive loads in the joint, at least not loads that are detectable with the range of Fujifilm used and the methods employed [55]. Even if ligament transection were to render the meniscus unstable and alter the contact area and pressure, the effect would be to decrease the contact area and increase the pressure, tending to minimise the potential difference between the intact, torn, and repaired knee menisci. However, care is required to keep the main stabilising elements of the knee, the cruciate and collaterals, intact. Otherwise, this creates rotational instability within the knee, affecting the pressure readings of the sensor. Only one included study incised the collateral for pressure sensor insertion [27]. Interestingly, this was one of the very few that did not restore TFCM following meniscal root repair.

Frequent calibration checks can minimise pressure measurement errors. Where described, Tekscan sensor studies had specific calibration protocols. This included having a maximum detection limit, an activation load for data collection, and tests being performed immediately after calibration. The latter is particularly significant in optimising sensitivity for force detection [56,57]. However, the calibration process, though simple, can be exhaustive and repetitive.

Capacitive sensors do not require re-calibration [29,32,35], and thus, they may be more useful for complex and serial experiments where several testing conditions are under investigation. Furthermore, capacitive sensors are shown to better conform to shaped surfaces and have a lower detection error [58]. In addition, their higher repeatability means they are less likely to be affected by sensor re-position compared to the Tekscan system. However, their 1 mm dielectric thickness results in more uniform redistribution of loading, which may not represent the actual loading of the joint being measured [12].

Fujifilm (Tokyo, Japan) is static, passive technology whereby its colour intensity is proportional to the applied load (colour-based sensors). Pressure can be applied to the films in one of two ways: (a) gradually increasing the pressure to the required level in two minutes and maintaining that pressure for another two minutes, which is known as continuous pressure, or (b) applying pressure for five seconds and maintaining pressure for another five seconds, which is known as momentary pressure. For ±10% precision, the recommended temperature and room humidity range is 20–35°C and 35–80%, respectively. After loading, the imprinted Fujifilm is usually photographed and imported into a customised MATLAB programme (Mathworks, Natwick, Massachusetts) based on the optical density of the scanned Fujifilm and a fifth-order polynomial developed from Fuji film calibration data. The calibration data are provided in the manufacturer guidelines, with each pressure film having its own pixel values and colour density. A written coded file converts this intensity to stress, providing an estimate of the local contact stress and area. Although it only provides a measure of TFCM at one time under one set of circumstances [58,59,60], its use with instantaneous loads can help to avoid the redistribution of fluid in articular cartilage and, in turn, pressure distributions that occur when testing a joint with a static load [61]. There are various ranges of films available (super low–super high), all of which operate under different load levels to ensure pressure maps can be created without the film becoming fully saturated. This may limit the acquisition of data based on the load applied. However, the super-low-range film (0.5–2.5 MPa) has greater pressure resolution compared to its counterparts [62], and Martens et al. noted a load of 1.75× body weight provides accurate pressure maps under these circumstances [55]. This was further underlined in the three Fujifilm studies in this review [1,28,39]. All provided appropriate pressure maps and contact area with the super-low film at loads between 1000 and 1800 N, reflecting the calculated target described by Martens et al. [55]. Harris et al. reported that although Fujifilm underestimated the contact area compared to Tekscan sensors across all flexion angles [63], this difference was reduced with the super-low films, particularly at 1000 N, the commonest load for biomechanical testing in this review. However, its relatively lower level of accuracy is suggestive in the use of the alternative sensors described. Yet despite this, underestimation of TFCM with Fujifilm occurred across all testing conditions, therefore minimising the potential difference between the intact, torn, and repaired meniscal root. In fact, all three studies showed similar conclusions to those of Tekscan and capacitive sensors [1,28,39], whereby they detected TFCM to restore to the intact state following root repair. In this context, super-low Fujifilm, both cheap and readily accessible, may be a viable alternative to the more desirable options in situations where simpler, more static, and preliminary experiments are undertaken.

### Limitations

This review is not without limitations. The presence of a variety of surgical techniques, loading cycles, and performance loads may have altered the true TFCM and its detection by the sensors, thereby confounding the biomechanical outcomes. However, similar conclusions in many of the included studies suggest these effects were minimal. Furthermore, we included porcine knees, which do not represent the consequences of a posterior meniscal root tear and repair to that of a human knee. Nevertheless, they are considered a valid alternative in the orthopaedic field, especially for meniscal root studies, due to the analogous function between the model and the anatomy of the human knee with persistent material properties [14,64,65]. Thus, their structural and mechanical properties were unlikely to have affected the pressure sensors. Finally, as all were time-zero studies, the pressure sensors could not accurately reflect the true behaviour of the meniscus, particularly once it has healed. Care should therefore be taken to extrapolate such findings in clinical practice, particularly as the sensors do not account for biological factors such as soft-tissue status, muscle contractions, proprioception, healing, and cartilage status. However, this limitation is more in keeping with the study’s methodological design than the sensor itself. There is no study to date comparing the common sensors described in this review on posterior meniscal root tears. Whilst one study in knee arthroplasty has compared Tekscan and Fujifilm [63], demonstrating more reliable and reproducible measurements with the Tekscan system, it is likely these results would correlate in root repair experiments. However, this requires further investigation to truly identify their differences in performance comparison to select the most suitable sensor.

Whilst most of the studies (21 out of 24) showed root repair to restore TFCM to that of the intact state, none were able to provide an analysis regarding how the repair directly influences the way the sensor collects the data. This requires further exploration.

The effect these laboratory data have on clinical implications is yet to be explored. Whilst wearable inertial measurement units and force-sensing sensors are used in practice to estimate joint kinematics and reaction force [66,67], introducing Tekscan, Fujifilm, or capacitive sensors within a real-life knee joint for dynamic simulation and loading is practically impossible. However, from a biomechanical perspective, the sensors do indirectly provide surgeons with evidence to argue for or against performing such repair techniques in clinical practice, particularly as the overall certainty of evidence from this review is high.

Further limitations are that majority of the studies did not provide in-depth analysis regarding how the sensors were able to detect pressure gradients, the computing of contact force, and the movement of the centre of pressure in flexion. Although this may have been beyond the scope of description for each individual study, such information would provide the reader an idea of the simplicity or complexity of the mathematical algorithm and coding sequences required to provide outcome data for the tibiofemoral contact mechanics.

## 5. Conclusions

Piezoresistive Tekscan and digital capacitive sensors are preferred for the collection of data during dynamic simulation of joint movements in intact, torn, and repaired posterior meniscal roots. Understanding the potential factors that influence such variables may improve accuracy, area, and pressure distribution measurements. The colour-based Fujifilm sensor is a passive technology that is ideal in simpler experiments with fewer cycles of load application.

## Figures and Tables

**Table 1:** Biomechanical characteristics of included studies. ACL, anterior cruciate ligament; ATPR, anatomic transtibial pull-through; HCT, horizontal cleavage tear; LFC, lateral femoral condyle; LM, lateral meniscus; LMPHT, lateral meniscal posterior horn tear; LMPRT, lateral meniscus posterior root tear; MAT, meniscal allograft transplantation; MD, mean difference; MM, medial meniscus; MMPH, medial meniscus posterior horn; MMPRT, medial meniscus posterior root tear; MCL, medial collateral ligament; MFC, medial femoral condyle; MFL, menisco-femoral ligament; MTL, meniscotibial ligament; MTM, material-testing machine; NATPR, non-anatomic transtibial pull-through; PCL, posterior cruciate ligament; PMMA, polymethylmethacrylate; TFCM, tibiofemoral contact mechanics; TPR, transtibial pull through repair. Biomechanical characteristics of included studies. ASA, all suture anchor; MMA, modified Mason–Allen; TSS, two simple stitch.

Study	Aims of Study	Outcome Measure of Interest (Effect Measures, EM)	Knee Specimen	No. of Specimens	Specimen Preparation	Bone Set Up
Baratz et al., 1986 [28]	Compare TFCM between intact MM and LM, arthroscopic and open MM tear repair partial and total meniscectomy	Contact area and pressure (EM: not reported)	Human cadaveric	3	1. 5 cm cut femur and tibia from joint 2. Tissue dissection with preservation of knee stabilisers	1. Femur and tibia potted in aluminium sleeves with screws
Allaire et al., 2008 [1]	Compare TFCM between intact meniscus, MMPRT, MMPRT repair, and total medial meniscectomy	Contact area and pressure (EM: MD)	Human cadaveric	9	1. Stored at −20 °C 2. Tissue dissection with preservation of knee stabilisers	1. Femur and tibia potted in resin 2. Mounted on MTM via jig
Marzo et al., 2008 [3]	Compare TFCM between intact meniscus, MMPH avulsion, and MMPH avulsion repair	Contact area and pressure (EM: not reported)	Human cadaveric	8	1. Femur and tibia cut from joint 2. Tissue dissection with preservation of knee stabilisers	1. Tibia and femur potted in PMMA 2. Distal femur attached to MTM through a transepicondylar rod
Seo JH et al., 2009 [21]	Compare TFCM between intact meniscus, MMPRT, and MMPRT repair	Contact area and pressure (EM:MD)	Porcine	11	1. Stored at −20 °C 2. 20 cm cut femur and tibia from joint 3. Tissue dissection with preservation of knee stabilisers	1. Femur and tibia potted in resin 2. Mounted on MTM via jig that enabled 6° of freedom
Muriuki et al., 2011 [39]	Compare TFCM between intact MM, radial and vertical tears, repair of tears, and total medial meniscectomy	Contact area and pressure (EM: MD)	Human cadaveric	11	1. 20 cm cut femur and tibia from joint 2. Tissue dissection with preservation of knee stabilisers	1. Femur and tibia potted in resin 2. Mounted on MTM via jig
Schillhammer et al., 2012 [46]	Compare TFCM between intact meniscus, LMPHT, and LMPHT repair	Contact area and pressure (EM: MD)	Human cadaveric	8	1. 15 cm cut femur and tibia from joint 2. Tissue dissection with preservation of knee stabilisers	Inadequate description
Kim JG et al., 2013 [35]	Compare TFCM between intact menisci, MMPRT, MMPRT repair, MAT, and MAT + MCL release	Contact area and pressure (EM: MD)	Human cadaveric	5 paired (10)	1. Stored at −25 °C 2. 15 cm cut femur and tibia from joint 3. Skin, fat, and muscle dissection	1. Femur and tibia potted in PMMA 2. Mounted on MTM via jig
Forkel et al., 2014 [32]	Compare TFCM between intact meniscus, LMPRT + intact MFL, LMPRT + MFL injury, and LMPRT repair with MFL injury via either traditional or ACL tunnel	Contact pressure (EM: MD)	Human cadaveric	10	1. Stored at −25 °C 2. Skin, fat, and muscle dissection	1.Tibia potted in plastic 2. Mounted on MTM via clamps
LaPrade CM et al., 2014 [38]	Compare TFCM between intact meniscus, LMPRT, and radial tear close to root, and the repair of these pathologies	Contact area and pressure (EM: MD)	Human cadaveric	8	1. 20 cm cut femur and tibia/fibula from joint 2. Tissue dissection with preservation of knee stabilisers	1. Tibia potted in PMMA 2. Distal femur attached to MTM through a transepicondylar rod 3. Proximal parallel femoral rod for angle adjustments 4. LFC osteotomy
Padalecki et al., 2014 [40]	Compare TFCM between intact meniscus and radial tears 3, 6 and 9 mm from MM root, and the repair of these pathologies	Contact area and pressure (EM: MD)	Human cadaveric	6	As per LaPrade CM 2014 [38]	As per LaPrade CM 2014 [38]
LaPrade CM et al., 2015 [37]	Compare TFCM between intact meniscus, MMPRT, MMPRT + ACL tear, ATPR for MMPRT, and NATPR for MMPRT	Contact area and pressure (EM: MD)	Human cadaveric	6	As per LaPrade CM 2014 [38]	As per LaPrade CM 2014 [38]
Perez-Blanca et al., 2015 [43]	Compared TFCM between intact meniscus, LMPRT, LMPRT repair, and meniscectomy	Contact area and pressure (EM: MD)	Human cadaveric	8	1. Stored in freezer 2. 12 cm cut femur and tibia from joint 3. Tissue dissection with preservation of knee stabilisers	1. Femur and tibia potted in resin 2. Mounted on MTM via clamps
Geeslin et al., 2016 [33]	Compare TFCM between intact meniscus, LMPRT + intact MFL, LMPRT + deficient MFL, LMPRT + deficient MFL + ACL tear, and LMPRT repair with ACL reconstruction + deficient MFL	Contact area and pressure (EM: MD)	Human cadaveric	10	1. Tissue dissection with preservation of knee stabilisers (except for group requiring MFL and/or ACL tear)	As per LaPrade CM 2014 [38]
Koh et al., 2016 [36]	Compare TFCM between intact meniscus, HCT of MM extending to root, inferior leaf resection, inferior and superior leaf resection, and HCT repair	Contact area and pressure (EM: MD)	Human cadaveric	12	1. 15 cm cut femur and tibia from joint 2. Tissue dissection with preservation of knee stabilisers	1. Distal femur attached to MTM via TEA rod 2x femoral tunnels 2. Proximal parallel femoral rod for angle adjustments 3. Tibia potted in cylinder with screws 4. MFC osteotomy
Chung et al., 2018 [29]	Compare TFCM between intact meniscus, MMPRT, and various MMPRT repair techniques	Contact area and pressure (EM: MD)	Porcine	7	1. 20 cm cut femur and tibia from joint 2. Tissue dissection with preservation of knee stabilisers	1. Femur and tibia potted with resin. 2. Femur mounted on MTM via jig that enabled 6° of freedom
Daney et al., 2019 [30]	Compare TFCM between intact meniscus, MMPRT, and various techniques for MMPRT repair	Contact area and pressure (EM: MD)	Human cadaveric	10	1. Stored in freezer, 2. 15–20 cm cut femur tibia from joint 3. Tissue dissection with preservation of knee stabilisers	1. As per LaPrade CM 2014 [38] 2. MFC osteotomy
Saltzman et al., 2020 [45]	Compare TFCM between intact meniscus, MMPRT, and MMPRT repair with PCL suture fixation	Contact area and pressure (EM: MD)	Human cadaveric	8	1. 20 cm cut femur and tibia/fibula from joint 2. Skin, fat, and muscle dissection	1. Femur and tibia potted in PMMA 2. Tibia mounted on custom-made table 3. Femur mounted on MTM via jig
Zhang et al., 2021 [47]	Compare TFCM between 4 repair techniques for T2 LMPRT: TPR, SAR, side-to-side repair, and H-plasty repair	Contact area and pressure (EM: MD)	Human cadaveric	24	1. Stored at −80 °C 2. 15 cm cut femur and tibia from joint 3. Tissue dissection with preservation of knee stabilisers	1. Femur and tibia potted with PMMA 2. Mounted on MTM via clamps 3. LFC osteotomy
Gupta et al., 2022 [34]	Compare TFCM between intact meniscus, LMPRT, and LMPRT repair	Contact area and pressure (EM: MD)	Human cadaveric	8	1. Stored at −20 °C 2. Tissue dissection with preservation of knee stabilisers	1. Femur and tibia potted in PMMA 2. Mounted on MTM via clamps 3. LFC osteotomy
Amano et al., 2023 [27]	Compare TFCM between intact meniscus, MMPRT, and various techniques for MMPRT repair	Contact area and pressure (EM: MD)	Porcine	10	1. Stored in freezer 2. 4–7 cm cut femur and tibia from joint 3. Skin, fat, and muscle dissection	1. Femur and tibia potted in PMMA 2. Mounted on MTM via jig
Doan et al., 2023 [31]	Compare TFCM between intact meniscus, MTL tear, MTL tear + MMPRT, MTL tear and MMPRT repair, and MTL tenodesis + MMPRT repair	Contact area and pressure (EM: MD)	Human cadaveric	10	1. Stored in freezer 2. 15–20 cm cut femur and tibia from joint 3. Tissue dissection with preservation of knee stabilisers	1. Femur and tibia potted in PMMA 2. Mounted on MTM via jig 3. MFC osteotomy
Park HJ et al., 2023 [41]	Compare TFCM between intact meniscus, MMPRT, and MMPRT repair. All were compared with varying degrees of OWHTO (neutral, 5°, and 10° of valgus).	Contact area and pressure (EM: MD)	Human cadaveric	9	1. 20–25 cm cut femur and tibia from joint	1. Femur and tibia potted in resin 2. Mounted on MTM via jig
Pasic et al., 2023 [42]	Compare TFCM between intact menisci, MMPRT, and repair techniques for MMPRT: TPR and all-inside repair	Contact pressure (EM: MD)	Human cadaveric	9	As per LaPrade CM 2014 [38]	As per LaPrade CM 2014 [38]
Saengpetch et al., 2023 [44]	Compare TFCM between intact meniscus, MMPRT, and repair techniques for MMPRT: TPR and all-suture anchor	Contact area and pressure (EM: MD)	Porcine	20	1. 20 cm cut femur and tibia from joint 2. Tissue dissection with preservation of knee stabilisers	1. Femur and tibia mounted on MTM via jig
**Study**	**Biomechanical Testing**	**Meniscal Tear Creation**	**Repair**		**Type of Pressure Sensor Used**	**Conclusions**
Baratz et al., 1986 [28]	Instron 1122 (Instron, MA, USA) 1. 1.8 kN load, 6 s cycle at 0°	Peripheral tear: 1. 2 cm MMPH or LMPH	**Arthroscopic repair**: 2 x horizontal mattress **Open repair:** 2 x vertical sutures		Colour-based sensor: Fujifilm Prescale (Tokyo, Japan)	**Peripheral tear**: Increased contact pressure compared to intact ** Post-repair:** TFCM restored to intact state
Allaire et al., 2008 [1]	TTS-25kN MTM (Ontario, Canada) 1. 1000 N load: 0, 30, 60, and 90° knee flexion	Not described	TPR		Colour-based sensor: Fujifilm Prescale (Tokyo, Japan)	**MMPRT**: Increased contact pressure compared to intact **Post-repair:** Pressure restored to intact state
Marzo et al., 2008 [3]	858 Mini Bionix MTM (Minneapolis, MA, USA) 1. 1800 N load at 0°	Posterior horn tear: At tibial attachment	TPR		Piezo-resistive sensor: Tekscan (Boston, MA, USA)	**MMPH tear**: Increased contact pressure and decreased area compared to intact **Post-repair:** TFCM restored to intact state
Seo JH et al., 2009 [21]	858 Mini Bionix MTM (Minneapolis, MA, USA) 1. 1500 N load: 0, 15, 30, 60, and 90° knee flexion	MMPRT: 3 mm from insertion site	TPR		Piezo-resistive sensor: K-Scan 6900 (Boston, MA, USA)	**MMPRT:** Increased contact pressure and decreased area to intact state at all angles **Post-repair**: Despite improvement in TFCM torn at most angles, only restored pressure to intact between 30 and 90°
Muriuki et al., 2011 [39]	MTM 1. 1000 N load: 0, 30, 60, and 90° knee flexion	**Radial tear:** MM central portion to inner rim ** Vertical tear:** Peripheral 1/3 involving root	**Radial repair: arthroscopic.** Horizontal mattress. ** Vertical repair:** Arthroscopic, vertical sutures		Colour-based sensor: Fujifilm Prescale (Tokyo, Japan)	**Radial tears**: No change in TFCM compared to intact state ** Vertical tears:** Increased contact pressure and reduced area, similar to total meniscectomy ** Post-repair:** TFCM in vertical tears resolved, similar to intact state
Schillhammer et al., 2012 [46]	Servohydraulic frame Load after 4th gait cycle	Insufficient description	TPR		Piezo-resistive sensor: Tekscan (Boston, MA, USA)	**LMPH detachment**: increased contact pressure and decreased area to intact state **Post-repair:** TFCM restored to intact state
Kim JG et al., 2013 [35]	Instron 8511 (Minneapolis, MN, USA) 1. 300 N load: 0, 30, 60, and 90° knee flexion	**Root tear:** Resection at tibial attachment	TPR		Capacitive sensor: Pliance X (Munich, Germany)	**MMPRT**: Increased contact pressure and decreased area compared to intact at mid-flexion angles ** Post-repair:** Improvement in TFCM to intact state
Forkel et al., 2014 [32]	Z005 (Ulm, Germany) 1. 100 N axial load at 0°	Insufficient description	TPR		Capacitive sensor: st Sensor Type S2042 (Munich, Germany)	**LMPRT**: Non-significant increase in contact pressure to intact **LMPRT + deficient MFL:** Significant increase in contact pressure to intact **Post repair:** Pressure restored to intact
LaPrade CM et al., 2014 [38]	Instron E10000 (Norwood, MA, USA) 1. 1000 N load: 0, 30, 45, 60, and 90° knee flexion	**Root tear:** Posterior root avulsion ** Radial tear:** 3 and 6 mm from root attachment	TPR		Piezo-resistive sensor: Tekscan (Boston, MA, USA)	**LMPRT and radial tear to root**: Increased contact pressure and decreased area compared to intact at all angles ** Post-root repair:** TFCM restored to intact state all angles
Padalecki et al., 2014 [40]	Instron E10000 (Norwood, MA, USA) 1. 1000 N axial load for 30 s: 0, 30, 45, 60, and 90° knee flexion	**Radial tear:** 3, 6, and 9 mm resection from root attachment ** Root tear:** At root attachment	TPR		Piezo-resistive sensor: Tekscan (Boston, MA, USA)	**MMPRT and radial tear:** Increased contact pressure and decreased area compared to intact at all angles **Post repair:** TFCM restored to intact state at all angles
LaPrade CM et al., 2015 [37]	Instron E10000, (Norwood, MA, USA) 1. 1000 N load for 30s: 0, 30, 45, 60, and 90° knee flexion	**MMPRT:** At root attachment	TPR (anatomical and non-anatomical)		Piezo-resistive sensor: Tekscan (Boston, MA, USA)	**MMPRT**: Increased contact pressure and decreased area to intact at all angles **Post NATPR**: No improvement in area to intact at all angles. Higher pressure to intact at 0 and 90°. ** Post ATPR:** TFCM restored to intact across all angles
Perez-Blanca et al., 2015 [43]	1. 1000N load: 0, 30, 60, and 90° knee flexion	Insufficient description	TPR		Piezo-resistive sensor: K-Scan 6900 (Boston, MA, USA)	**LMPRT and meniscectomy**: Increased contact pressure and decreased area to intact all angles ** Post-repair:** TFCM restored to intact state only at lower angles
Geeslin et al., 2016 [33]	Instron E10000, (Norwood, MA, USA) 1. 1000 N load for 30 s: 0, 30, 45, 60, and 90° knee flexion	**Root tear**: Tear between posterior root attachment and medial tibial eminence	TPR		Piezo-resistive sensor: Tekscan (Boston, MA, USA)	**LMPRT + intact MFL + intact ACL**: Similar TFCM to intact meniscus all angles **LMPRT + deficient MFL** (regardless of ACL condition): Increased contact pressure and decreased area to intact state all angles **LMPRT repair + ACL Reconstruction**: Similar TFCM to meniscus intact across most angles
Koh et al., 2016 [36]	1. 800 N load: 0 and 60°	**HCT tear**: 1 cm away from posterior root and extending into root	HCT repair: Inside out with 2 x vertical mattress		Piezo-resistive sensor: Tekscan (Boston, MA, USA)	**HCT at root**: Similar TFCM to intact state **Post repair:** Similar TFCM to intact state
Chung et al., 2018 [29]	Instron 8,511 (Minneapolis, MN, USA) 1. 1000 N load: 0, 30, 60, and 90° knee flexion	**MMPRT**: 5 mm radial tear from root attachment	**Either: a. All-inside repair:** Fastfix anchors **b. TPR with TSS or c. TPR with MMA**		Capacitive sensor (Munich, Germany)	**MMPRT**: Increased contact pressure and decreased area to intact state all angles ** Post-repair with TSS, MMA or All inside:** Despite improvements of pressure and area to torn state, did not restore to intact at all angles **Between fixation groups**: Similar pressure but better area with MMA at all angles
Daney et al., 2019 [30]	Instron E10000, (Norwood, MA, USA) 1. 1000 N load for 30s: 0, 30, 45, 60, and 90° knee flexion	**MMPRT:** Adjacent to root attachment	TPR (ATPR or NATPR) Centralisation: Transtibial technique		Piezo-resistive sensor: Tekscan (Boston, MA, USA)	**MMPRT**: Increased contact pressure and decreased area to intact all angles ** ATPR repair:** TFCM restored to intact at all angles ** ATPR + centralisation:** TFCM similar to intact
Saltzman et al., 2020 [45]	858 Mini Bionix MTM (Minneapolis, MA, USA) 1. 1500 N load: 0, 30, 60, and 90°	**MMPRT**: 2–3 mm from tibial insertion	MMPRT repair with PCL suture fixation: Horizontal mattress and Mason Allen fixation		Piezo-resistive sensor: Tekscan (Boston, MA, USA)	**MMPRT**: Increased contact pressure and decreased area to intact at all flexion angles ** MMPRT repair with PCL suture fixation:** TFCM restored to intact state across all flexion angles
Zhang et al., 2021 [47]	BOSE Testing machine 1. 1000 N load: 0, 30, 60, and 90° knee flexion	**LMPRT:** 9 mm from root insertion	**Either a TPR repair, b. SAR**: corkscrew anchor, **c. side-toside repair**: fast-fix devices, or **d. H-plasty**: combination of side-to-side repair and vertical sutures		Piezo-resistive sensor: Tekscan (Boston, MA, USA)	**LMPRT**: Increased contact pressure and decreased area to intact at all angles ** H-plasty:** TFCM restored to intact all angles **S-S:** TFCM restored to intact 0 and 30° ** TPR and SAR:** TFCM not restored to intact across at all angles
Gupta et al., 2022 [34]	Kuka (Augsburg, Germany) Body weight load at 0, 30, and 60° knee flexion	**LMPRT**: Tibial attachment site	**TPR**		Piezo-resistive sensor: Tekscan (Boston, MA, USA)	**LMPRT**: Decreased contact area to intact state at mid-high angles (30–60°) **Post-repair**: Area improved to intact at high angles (>30°). Pressure improved at low angles (<30°)
Amano et al., 2023 [27]	1. 200 N at 30, 45, 60, and 90° knee flexion	**MMPRT**: 9 mm medial from root attachment	**TPR** **Centralisation:** Knotless anchors		Piezo-resistive sensor: Tekscan (Boston, MA, USA)	**MMPRT**: Increased contact pressure and decreased area to intact at all angles ** TPR:** No restoration in TFCM to intact at all angles **NATPR + centralise:** TFCM similar to intact except at mid-flexion
Doan et al., 2023 [31]	E10000 Instron	**MTL tear:** Incision of distal fibres of the medial MTL insertion on tibia **MMPRT:** Radial tear 3 mm from insertion from centre of enthesis	**MTL repair**: 2 × 2.6 mm knotless anchor (FiberTak) TPR		Piezo-resistive sensor: K-Scan 4000 (Boston, MA, USA)	**MTL and root tear:** Increased contact pressure and decreased area to intact state ** MTL tear and root repair:** Similar TFCM to intact **MTL tenodesis and root repair:** Similar TFCM to intact
Park HJ et al., 2023 [41]	Instron 5567 (Norwood, MA, USA) 1. 650 N at 0°	**MMPRT:** ransection at bony attachment site	TPR		Piezo-resistive sensor: Tekscan (Boston, MA, USA)	**MMPRT:** Increased contact pressure and decreased area to intact state ** MMPRT repair:** TFCM restored to intact state
Pasic et al., 2023 [42]	Instron E10000, (Norwood, MA, USA) 1. 1000 N load at 0° knee flexion	**MMPRT:** Full-thickness radial tear	**Either a. all-inside repair**: 3x horizontal mattress or **b. TPR**		Piezo-resistive sensor: Tekscan (Boston, MA, USA)	**MMPRT**: Increased contact pressure compared to intact state ** Post AI repair (either):** TFCM restored to intact state
Saengpetch et al., 2023 [44]	Instron E10000, (Norwood, MA, USA) 1. 600 N load: 0, 45, and 90° knee flexion	**MMPRT**: 5 mm medial to tibial attachment	**Either a. TPR** or **b. all-suture anchor**: Y-knot anchor with MMA configuration		Piezo-resistive sensor: Tekscan (Boston, MA, USA)	**MMPRT**: Increased contact pressure and decreased area to intact state at all angles ** Post ASA:** Similar TFCM to intact all angles ** Post TPR:** Similar TFCM to intact except with area at 45° and pressure at 90°

**Table 2:** Pressure sensor application and insertion. N/A, not applicable; NR, not reported; kPA, kilo Pascals.

Study	Insertion of Sensor				Saline Soak		
	Arthrotomy	Preservation of Cruciate & Collateral Ligaments	Security		Pre-Testing	During Testing	Precautions Taken to Avoid Measurement Error in Saline
Baratz et al., 1986 [28]	2 cm incision in anterior meniscal attachment, Fujifilm positioned beneath meniscus	Yes	Insufficient detail		No, packaged in heat-sealed cellophane	No, fluid proof packet	No
Allaire et al., 2008 [1]	4x < 2 cm sub-meniscal arthrotomy, Fujifilm positioned beneath meniscus	Yes	Insufficient detail		Not described	No, fluid proof packet	No
Marzo et al., 2008 [3]	Incision in MF and MT ligament	Yes	Insufficient detail		Not described	Not described	No
Seo JH et al., 2009 [21]	Incision posterior MT ligament, Tekscan positioned beneath meniscus	Yes	Held in position by tightening capsule		Not described	Yes	No
Muriuki et al., 2011 [39]	4x < 2 cm sub-meniscal arthrotomies, Fujifilm positioned beneath meniscus	Yes	Insufficient detail		No, sealed to prevent moisture 3 days prior to test date	No, fluid proof packet	No
Schillhammer et al., 2012 [46]	Incision of coronary ligament, Tekscan positioned beneath meniscus	Yes	Tibial fixation with suture anchor		Not described	Not described	No
Kim JG et al., 2013 [35]	3 cm capsulotomy medial to PT and PCL, sensor positioned above meniscus	Yes	Insufficient detail		Not described	Not described	No
Forkel et al., 2014 [32]	Sensor positioned between the femur and meniscotibial surface in lateral joint space	Inadequate description	Insufficient detail		Not described	Not described	No
LaPrade CM et al., 2014 [38]	2x incisions in anterior and posterior MT ligament, Tekscan positioned beneath meniscus	Yes	Tibial fixation with suture anchor		Yes—48 h	Yes	Slight linear decline in load output countered by normalising data with measured linear rate of decline
Padalecki et al., 2014 [40]	Yes, per LaPrade 2014 [38]	Yes	Tibial fixation with suture anchor		Yes—48 h	Yes	Yes, per LaPrade 2014 [38]
LaPrade CM et al., 2015 [37]	Yes, per LaPrade 2014 [38]	Yes	Tibial fixation with suture anchor		Yes—48 h	Yes	Yes, per LaPrade 2014 [38]
Perez-Blanca et al., 2015 [43]	1x incision coronary ligament, sensor positioned beneath meniscus	Yes	Tibial fixation with screws		Not described	Not described	No
Geeslin et al., 2016 [33]	Yes, per LaPrade 2014 [38]	Yes	Tibial fixation with suture anchor		Yes—48 h	Yes	Yes, per LaPrade 2014 [38]
Koh et al., 2016 [36]	2x sub-meniscal arthrotomies anteriorly and posteriorly, Tekscan positioned beneath meniscus	Yes	Insufficient detail		Not described	Yes	No
Chung et al., 2018 [29]	Yes, per LaPrade 2014 [38]	Yes	Fixed to meniscocapsular junction		Not described	Yes	No
Daney et al., 2019 [30]	Yes, per LaPrade 2014 [38]	Not described	Tibial fixation with suture anchor		Not described	Yes	Yes, per LaPrade 2014 [38]
Saltzman et al., 2020 [45]	Yes, per LaPrade 2014 [38]	Not described	Tibial fixation with screws		Yes—48 h	Yes	No
Zhang et al., 2021 [47]	Yes, per LaPrade 2014 [38]	Yes	Fixed to surrounding ligamentous structures		Not described	Yes	No
Gupta et al., 2022 [34]	Yes, per LaPrade 2014 [38]	Yes	Insufficient detail		Not described	Yes	No
Amano et al., 2023 [27]	MCL sectioned, Tekscan positioned above meniscus	No, MCL sectioned	Insufficient detail		Not described	Yes	No
Doan et al., 2023 [31]	Yes, per LaPrade 2014 [38]	Yes	Tibial fixation with screws		Not described	Not described	No
Park HJ et al., 2023 [41]	Yes, per LaPrade 2014 [38]	Yes	Tibial fixation with screws		Yes—48 h	Yes	No
Pasic et al., 2023 [42]	2x incision with one posterior to LCL and one posterior to MCL, sensor positioned beneath meniscus	Yes	Insufficient detail		Not described	No	No
Saengpetch et al., 2023 [44]	No sub-meniscal or capsular arthrotomy, sensor placed above meniscus	Yes	Fixed to knee joint soft tissue		Not described	Yes	No
**Study**	**Sensor Visualisation to Ensure Accurate Placement**	**Dealing with Wrinkling/Damage of Sensors**	**Pre-Tensioning**	**Sensor Calibration**			
				**Settings**	**Frequency**	**To Temperature**	**Cyclical Loading**
Baratz et al., 1986 [28]	Yes—through posterior arthrotomy	New Fujifilm for each test	N/A	N/A	N/A	Sensitive to temperature	N/A
Allaire et al., 2008 [1]	Yes—through posterior arthrotomy	New Fujifilm for each test	N/A	N/A	N/A	Fluid-proof packet to minimise °C changes	N/A
Marzo et al., 2008 [3]	Insufficient description	No description of whether new sensor used for each knee	No	Calibrated to 1800 N	No frequent calibration	NR	Not performed
Seo JH et al., 2009 [21]	Insufficient description	New sensor for each knee	Yes	Per manufacturer recommendations Calibrated to confirm 0 setting 0 to 15 MPa—test performed within this range	Between each test	NR	3 cycles of varying flexion angles for each meniscal condition
Muriuki et al., 2011 [39]	Insufficient description	New Fujifilm for each test	N/A	Not required	Not required	Fluid-proof packet to minimise °C changes	Not required
Schillhammer et al., 2012 [46]	Yes—disarticulation of femur for view	New sensor for each knee	No	Yes, calibrated using 3-point non-linear technique spanning lower pressure range to match experiment loads	Between knee specimens	NR	Yes, 5 gait cycles
Kim JG et al., 2013 [35]	Insufficient description	One sensor for all testing	Pre-tensioned 300 N	Preliminary testing to confirm 2500–3000 kPa safe and corresponded to 300 N	No re-calibration required	Yes	Cycled 3 times with a 300 N for pre-tensioning
Forkel et al., 2014 [32]	Yes, anterior horn lateral meniscus as reference	One sensor for all testing	No	Preliminary testing to confirm pressure up to 2000 kPA safe	No re-calibration required	Yes	Not performed
LaPrade CM et al., 2014 [38]	Yes, through LFC osteotomy	New sensor for each knee False measure replaced by mean of surrounding sensels	No	Per manufacturer’s recommendations No baseline reference reported	Between knee specimens	NR	Not performed
Padalecki et al., 2014 [40]	Yes, through MFC osteotomy	Per LaPrade 2014 [36]	No	Per manufacturer recommendations No baseline reference reported	Between knee specimens	NR	Not performed
LaPrade CM et al., 2015 [37]	Insufficient description	Per LaPrade 2014 [36]	No	Per manufacturer recommendations No baseline reference reported	Between knee specimens	NR	Not performed
Perez-Blanca et al., 2015 [43]	Insufficient description	New sensor for each knee	No	Per manufacturer recommendations No baseline reference reported	Between each test	NR	5 cycles of 1200 N
Geeslin et al., 2016 [33]	Yes, through LFC osteotomy	Per LaPrade 2014 [36]	No	Per manufacturer recommendations No baseline reference reported	Between knee specimens	NR	Not performed
Koh et al., 2016 [36]	Yes, through MFC osteotomy	Insufficient description	No	Calibrated to 800 N	Every 2 consecutive experiment	NR	Not performed
Chung et al., 2018 [29]	Insufficient description	One sensor for all testing	Yes, pre-tensioned	Calibrated to confirm 0 setting Preliminary testing confirmed pressure to 3200 kPA safe	No re-calibration required	Yes	Not performed
Daney et al., 2019 [30]	Yes, through MFC osteotomy	New sensor for each knee	No	Per manufacturer recommendations No baseline reference reported	Between knee specimens	NR	Not performed
Saltzman et al., 2020 [45]	Insufficient description	New sensor for each knee	Pre-tensioned 25 N	Per manufacturer recommendations Calibrated to confirm 0 setting	Between knee specimens	NR	Not performed
Zhang et al., 2021 [47]	Yes, through LFC osteotomy	One sensor for all testing	Pre-tensioned 200 N	Per manufacturer recommendations No baseline reference reported	Between each test	NR	3 cycles of varying knee flexion angles
Gupta et al., 2022 [34]	Yes, through LFC osteotomy	New sensor for each knee	No	Per manufacturer recommendations No baseline reference reported	Not described	NR	Not performed
Amano et al., 2023 [27]	Insufficient description	New sensor for each knee	Pre-tensioned 80 N	Per manufacturer recommendations No baseline reference reported	Between knee specimens	NR	Not performed
Doan et al., 2023 [31]	Yes, through MFC osteotomy	Insufficient description	No	Per manufacturer recommendations Calibrated to perform experiment within target range	Not described	NR	Not performed
Park HJ et al., 2023 [41]	Insufficient description	Insufficient description	No	Per manufacturer recommendations No baseline reference reported	Between knee specimens	NR	Not performed
Pasic et al., 2023 [42]	Yes—small load applied on joint and visually confirming contact area fully covered by sensor	Insufficient description	No	Per manufacturer recommendations No baseline reference reported	Between each test	NR	Not performed
Saengpetch et al., 2023 [44]	Insufficient description	Insufficient description	Yes	Calibrated to confirm 0 setting	Between each test	NR	Not performed

## Data Availability

No new data were created or analysed in this study.

## Supplementary Materials

The following supporting information can be downloaded at: https://www.mdpi.com/article/10.3390/s25051507/s1, Table S1. Search strategy for Pubmed (Medline), Embase and Cochrane Central Register of Controlled Trials (15 January 2024). Table S2. Search strategy for Pubmed (Medline), Embase and Cochrane Central Register of Controlled Trials (10 November 2024). Table S3. MINORS Quality Appraisal Tool. NOTE. Each item was assigned a score of 0 points (not reported), 1 point (reported but inadequate), or 2 points (reported and adequate). MINORS, Methodological Index for Non-randomized Studies. Table S4. Quality of Included Studies Assessed by MINORS Quality Appraisal Tool. NOTE. Each item was assigned a score of 0 points (not reported), 1 point (reported but inadequate), or 2 points (reported and adequate), with a maximum possible score of 24. Item 1 indicates a clearly stated aim; item 2, inclusion of consecutive patients; item 3, prospective collection of data; item 4, endpoints appropriate to the aim of the study; item 5, unbiased assessment of the study endpoint; item 6, follow-up period appropriate to the aim of the study; item 7, loss to follow-up less than 5%; item 8, prospective calculation of the study size; item 9, an adequate control group; item 10, contemporary groups; item 11, baseline equivalence of groups; and item 12, adequate statistical analyses. Figure S1. Risk of bias summary. Red circle, high risk of bias; yellow circle, moderate risk of bias; green circle, low risk of bias. D1: Bias due to confounding data (selection bias), D2: bias in selection of participants into the study (selection bias), D3: bias in classification of interventions (information bias), D4: bias due to deviations from intended interventions (performance bias), D5: bias due to missing data (attrition data), D6: bias in measurement of outcomes (detection bias), D7: bias in selection of the reported result (outcome reporting bias). Figure S2. Risk of Bias graph.

## Abbreviations

The following abbreviations are used in this manuscript:

ACLR	anterior cruciate ligament reconstruction
ATPR	anatomical transtibial pull-through repair
HCT	horizontal cleavage tear
LFC	lateral femoral condyle
LM	lateral meniscus
LMPHT	lateral meniscus posterior horn tear
LMPRT	lateral meniscus posterior root tear
MAT	meniscal allograft transplantation
MCL	medial collateral ligament
MFC	medial femoral condyle
MFL	meniscofemoral ligament
MTM	materials testing machine
MM	medial meniscus
MMA	modified Mason–Allen
MMPH	medial meniscus posterior horn
MMPRT	medial meniscus posterior root tear
MTL	meniscotibial ligament
NATPR	non-anatomic transtibial pull-through repair
OWHTO	open-wedge high tibial osteotomy
PCL	posterior cruciate ligament
PMMA	polymethylmethacrylate
PMRT	posterior meniscus root tear
PRISMA	Preferred Reporting Items for Systematic Review and Meta-Analyses
SAR	suture anchor repair
TFCM	tibiofemoral contact mechanics
TPR	transtibial pull-through repair
TSS	two simple stitch
