# Supplementary material for: Pressure Sensors for Measuring Tibiofemoral Contact Mechanics in Meniscal Root Repair: A Systematic Review
Source: Sensors (Basel). 2025 Feb 28;25(5):1507. doi: 10.3390/s25051507 (PMC11902513; doi:10.3390/s25051507)
Supplement: Supplementary file 1 [file sensors-25-01507-s001.zip › sensors-3451348-supplementary.pdf]

Supplementary Material

	Pubmed (Medline) (15th January 2024)	EMBASE (15th January 2024)	Cochrane Central Register of Controlled Trials (15th January 2024)
1	posterior horn.mp	posterior horn.mp	posterior horn.mp
2	root.mp	root.mp	root.mp
3	radial.mp	radial.mp	radial.mp
4	meniscus.mp	meniscus.mp	meniscus.mp
5	meniscal.mp	meniscal.mp	meniscal.mp
6	tibiofemoral.mp	tibiofemoral.mp	tibiofemoral.mp
7	contact area.mp	contact area.mp	contact area.mp
8	contact pressure.mp	contact pressure.mp	contact pressure.mp
	(1 OR 2 OR 3) AND (4 OR 5) AND (6 OR 7 OR 8)	(1 OR 2 OR 3) AND (4 OR 5) AND (6 OR 7 OR 8)	(1 OR 2 OR 3) AND (4 OR 5) AND (6 OR 7 OR 8)
	Results: 240	Results: 309	Results: 6

Table S1. Search strategy for Pubmed (Medline), Embase and Cochrane Central Register of Controlled Trials (January 15th 2024)

	Pubmed (Medline) (10th July 2024)	EMBASE (10th November 2024)	Cochrane Central Register of Controlled Trials (10th November 2024)
1	posterior horn.mp	posterior horn.mp	posterior horn.mp
2	root.mp	root.mp	root.mp
3	radial.mp	radial.mp	radial.mp
4	meniscus.mp	meniscus.mp	meniscus.mp
5	meniscal.mp	meniscal.mp	meniscal.mp
6	tibiofemoral.mp	tibiofemoral.mp	tibiofemoral.mp
7	contact area.mp	contact area.mp	contact area.mp
8	contact pressure.mp	contact pressure.mp	contact pressure.mp
	(1 OR 2 OR 3) AND (4 OR 5) AND (6 OR 7 OR 8)	(1 OR 2 OR 3) AND (4 OR 5) AND (6 OR 7 OR 8)	(1 OR 2 OR 3) AND (4 OR 5) AND (6 OR 7 OR 8)
	Results: 245	Results: 316	Results: 6

Table S2. Search strategy for Pubmed (Medline), Embase and Cochrane Central Register of Controlled Trials (10th November 2024)

Item	Description
1	A clearly stated aim: The question addressed should be precise and relevant in light of the available literature.
2	Inclusion of consecutive patients: All patients potentially fit for inclusion (satisfying the criteria for inclusion) have been included in<br>the study during the study period (with no exclusion or with details about the reasons for exclusion).
3	Prospective collection of data: Data have been collected according to a protocol established before the beginning of the study.
4	Endpoints appropriate to the aim of the study: An unambiguous explanation of the criteria used to evaluate the main outcome has been provided, which should be in accordance with the question addressed by the study. Also, the endpoints should be assessed on an intention-to-treat basis
5	Unbiased assessment of the study endpoint: Blind evaluation of objective endpoints and double-blind evaluation of subjective<br>endpoints has been performed. Otherwise, the reasons for not blinding should be stated.
6	Follow-up period appropriate to the aim of the study: The follow-up should be sufficiently long to allow the assessment of the main endpoint and possible adverse events.
7	Loss to follow-up < 5%: All patients should be included in the follow-up. Otherwise, the proportion lost to follow-up should not exceed the proportion experiencing the major endpoint.
8	Prospective calculation of the study size: Information has been provided on the size of detectable difference of interest with a calculation of the 95% confidence interval, according to the expected incidence of the outcome event, and information has been provided about the level of statistical significance and estimates of power when comparing the outcomes.
9	An adequate control group: A gold-standard diagnostic test or therapeutic intervention recognized as the optimal intervention according to the available published data has been used.
10	Contemporary groups: The control and study groups should be managed during the same period (no historical comparison).
11	Baseline equivalence of groups: The groups should be similar regarding the criteria other than the studied endpoints. There should be an absence of confounding factors that could bias the interpretation of the results.
12	Adequate statistical analyses: It should be determined whether the statistics were in accordance with the type of study with calculation of confidence intervals or relative risk.

Table S3. MINORS Quality Appraisal Tool.

NOTE. Each item was assigned a score of 0 points (not reported), 1 point (reported but inadequate), or 2 points (reported and adequate). MINORS, Methodological Index for Non-randomized Studies.

Score													
Authors (year)	Item 1	Item 2	Item 3	Item 4	Item 5	Item 6	Item 7	Item 8	Item 9	Item 10	Item 11	Item 12	Total	% of maximum score
Biomechanical Studies														
Baratz et al 1986 [28]	2	2	2	2	0	2	2	0	2	2	2	0	18	75
Allaire et al 2008 [1]	2	2	2	2	0	2	2	1	2	1	2	2	20	83.3
Marzo et al 2008 [3]	2	2	2	2	0	2	2	1	2	1	2	2	20	83.3
Seo JH et al 2009 [21]	2	2	2	2	0	2	2	1	2	1	2	1	19	79.2
Muriuki et al 2011 [39]	2	2	2	2	1	2	2	1	2	1	2	1	20	83.3
Schillhammer et al 2012 [46]	2	2	2	2	0	2	2	1	2	2	2	1	20	83.3
Kim JG et al 2013 [35]	2	2	2	2	0	2	2	1	2	1	2	1	19	79.2
Forkel et al 2014 [32]	2	2	2	2	1	2	2	1	2	2	2	2	22	91.2
LaPrade CM et al 2014 [38]	2	2	2	2	1	2	2	1	2	2	2	2	22	91.2
Padalecki et al 2014 [40]	2	2	2	2	1	2	2	1	2	2	2	2	22	91.2
LaPrade CM et al 2015 [37]	2	2	2	2	1	2	2	1	2	2	2	2	22	91.2
Perez-Blanca et al 2015 [43]	2	2	2	2	0	2	2	0	2	2	2	1	19	79.2
Geeslin et al 2016 [33]	2	2	2	2	1	2	2	1	2	2	2	1	21	87.5
Koh et al 2016 [36]	2	2	2	2	0	2	2	0	2	2	2	1	19	79.2
Chung et al 2018 [29]	2	2	2	2	0	2	2	0	2	2	2	1	19	79.2
Daney et al 2019 [30]	2	2	2	2	1	2	2	1	2	2	2	2	22	91.2
Saltzman et al 2020 [45]	2	2	2	2	1	2	2	1	2	2	2	2	22	91.2
Zhang et al 2021 [47]	2	2	2	2	0	2	2	0	2	2	2	1	19	79.2
Gupta et al 2022 [34]	2	2	2	2	1	2	2	1	2	2	2	2	22	91.2

Amano et al 2023 [27]	2	2	2	2	1	2	2	1	2	2	2	2	22	91.2
Doan et al 2023 [31]	2	2	2	2	1	2	2	1	2	2	2	1	21	87.5
Park HJ et al 2023 [41]	2	2	2	2	0	2	2	1	2	2	2	1	20	83.3
Pasic et al 2023 [42]	2	2	2	2	1	2	2	1	2	2	2	2	22	91.2
Saengpetch et al 2023 [44]	2	2	2	2	1	2	2	1	2	2	2	1	21	87.5

Table S4. Quality of Included Studies Assessed by MINORS Quality Appraisal Tool.

NOTE. Each item was assigned a score of 0 points (not reported), 1 point (reported but inadequate), or 2 points (reported and adequate), with a maximum possible score of 24. Item 1 indicates a clearly stated aim; item 2, inclusion of consecutive patients; item 3, prospective collection of data; item 4, endpoints appropriate to the aim of the study; item 5, unbiased assessment of the study endpoint; item 6, follow-up period appropriate to the aim of the study; item 7, loss to follow-up less than 5%; item 8, prospective calculation of the study size; item 9, an adequate control group; item 10, contemporary groups; item 11, baseline equivalence of groups; and item 12, adequate statistical analyses.

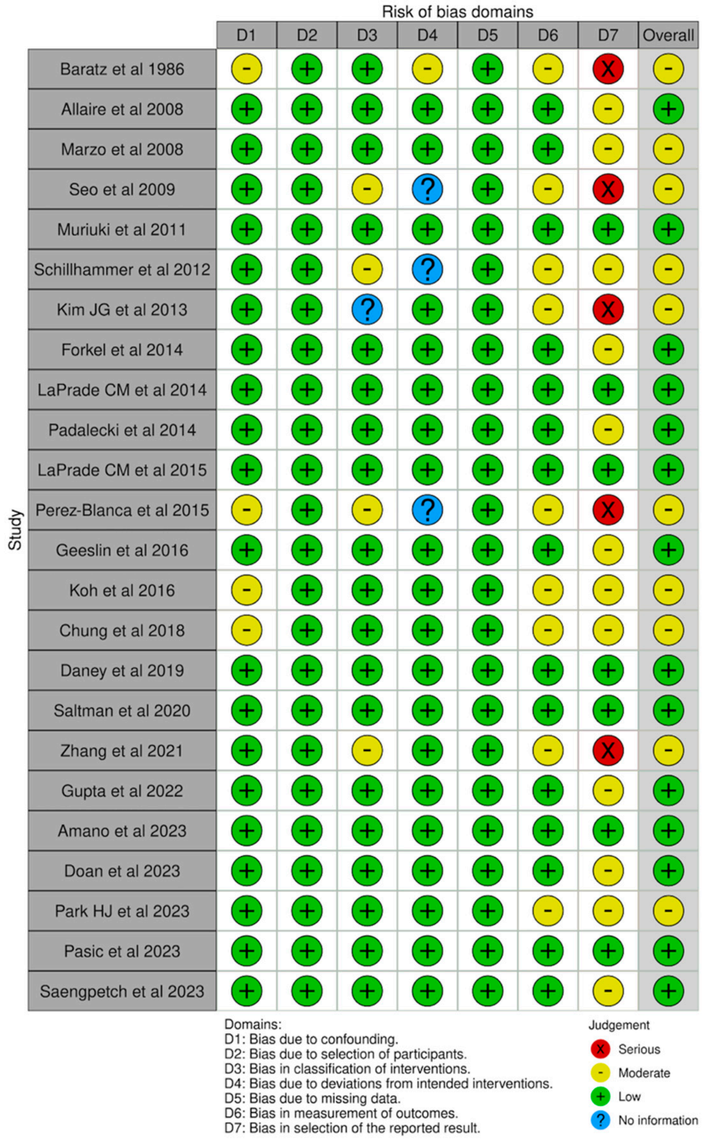

Figure S1. Risk of bias summary. Red circle, high risk of bias; yellow circle, moderate risk of bias; green circle, low risk of bias. D1: Bias due to confounding data (selection bias), D2: bias in selection of participants into the study (selection bias), D3: bias in classification of interventions (information bias), D4: bias due to deviations from intended interventions (performance bias), D5: bias due to missing data (attrition data), D6: bias in measurement of outcomes (detection bias), D7: bias in selection of the reported result (outcome reporting bias

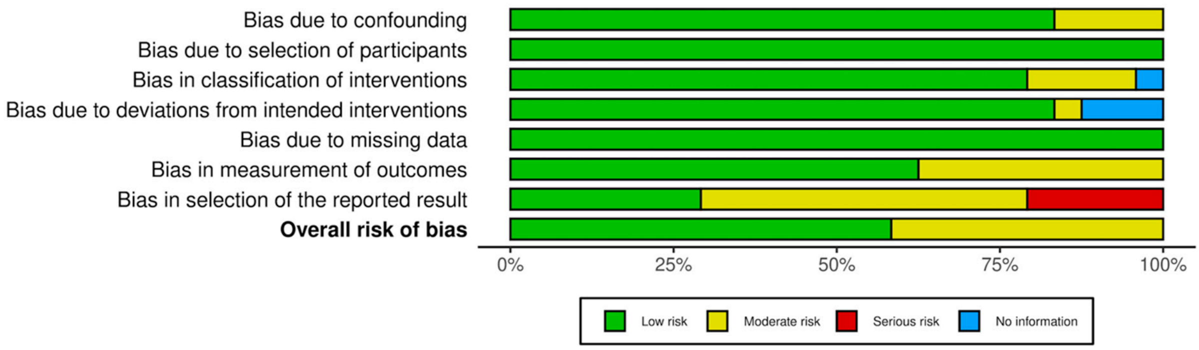

Figure S2. Risk of Bias graph
